# Proposal and Validation of a Measurement Scale of the Acceptance of Ultra-Processed Food Products

**DOI:** 10.3390/foods13101481

**Published:** 2024-05-10

**Authors:** Cristina Calvo-Porral, Sergio Rivaroli, Javier Orosa-González

**Affiliations:** 1 Business Department, Facultad Economía y Empresa, University of A Coruña, Campus Elviña s/n, 15006 A Coruña, Spain; javier.orosa@udc.es; 2 Dipartimento di Scienze e Tecnologie Agro-Alimentari, Alma Mater Studiorum, Universitá di Bologna, 40127 Bologna, Italy; sergio.rivaroli@unibo.it

**Keywords:** ultra-processed food, measurement scale, validation, consumption, behavior

## Abstract

Today, there is an increasing consumption of ultra-processed food products (UPFs), while more healthy options are available; however, there is no scale available that can adequately measure this phenomenon. In this context, the present study aims to develop and validate a measurement scale of the consumers’ acceptance of ultra-processed food products. Research data (*n* = 478) were analyzed using Exploratory Factor Analysis (EFA), followed by a Confirmatory Factor Analysis (CFA). The results confirm the validity of the proposed measurement scale comprising nine factors: the quality of ultra-processed food products, ability to save time, low affordable price, effortless preparation, convenience, hedonic nature, marketing strategies, satisfaction and purchase intention. The present study makes a noticeable contribution to food marketing, and food companies could consider these factors to design and commercialize ultra-processed foods.

## 1. Introduction

While there is no standard definition of ultra-processed foods (UPFs), the NOVA classification assigns an edible substance to the UPFs group when it is typically created by a series of industrial techniques and processes and is primarily for industrial use. Since the NOVA food classification system was defined, researchers have increasingly linked the concept of UPFs with higher commercial profits for the food industry over fresh or minimally processed foods [1]. Due to some attributes of these products, such as their affordability, convenience, hedonic nature and high availability, UPFs have become a prevalent food option among consumers [2].

UPFs include not only “junk food”, but also foods commercialized and marketed as healthy, such as light, vegan, organic, or gluten-free products and include food categories such as chocolates, candy, mass-produced packaged breads, pastries, cookies, instant soups or sausages. These food products are characterized by highly processed ingredients and additives, have low nutritional value, a large amount of energy, refined carbohydrates, sodium, trans fat and saturated fatty acids [1]. For this reason, UPFs are frequently associated with unhealthful food products with high fat, sugar and salt content [2], but consumers increasingly demand and consume them, despite their negative image. On the other hand, governments and international organizations increasingly demand healthier and more natural food products from the food industry [3,4].

However, to the best of the authors’ knowledge, no scale was available to measure the phenomenon of consumer acceptance of UPFs. Considering that previous studies overlooked the proposal and development of a scale that could measure the acceptance and demand of UPFs, this study aims to cover this research gap and provide consumer behavioral researchers with a measurement tool to determine an individual’s attitude and acceptance towards UPFs.

So, the present study aims to answer the following research question: “is it possible to develop a measurement scale on the acceptance and consumption of UPFs?”, and if so, “what dimensions could be included in the measurement scale on the acceptance and consumption of UPFs?”. For this purpose, the present study develops a literature review, followed by an item and content development carried out by a group of experts and then statistically and empirically tests and validates a scale to measure the acceptance and consumption of UPFs. Therefore, the major contribution of the present research is the proposal of a new measurement scale for the acceptance of UPFs.

## 2. Conceptual Background

### 2.1. The Concept and Consumption of UPFs

The term UPFs appears in a research article by [5], whereby they define UPFs as “formulations of ingredients, mostly of exclusive industrial use, that result from a series of industrial processes” [6]. However, nowadays there is a lack of legal norms defining what a UPF is, and in this context, there are several definitions proposed by different classification systems.

But, what is the meaning of food processing? Interestingly, food processing was originally developed to resolve the problems related to food transport and long-time storage, whereas today, food processing is mostly focused on improving the palatability and production of indulgent food products [7]. According to [8,9], food processing could be defined as the transformation of ingredients into products or any modification of food that is subjected to altering its sensory quality or shelf life. Similarly, the concept of food processing could be also understood as the required steps in order to obtain a food product from raw materials, which involves a physical or chemical transformation of a food product [10]. According to the European Food Information Council (EUFIC), food processing consists of any method used to transform fresh food into a product, which includes adding components to the food to extend the shelf life, to retain nutritional and sensory quality, or to increase the nutritional quality.

The NOVA classification proposed a systematic and comprehensive review of UPFs, and divided these food products into four groups according to the type of industrial processing: (1) unprocessed and minimally processed foods; (2) processed culinary ingredients; (3) processed foods; and (4) “ultra-processed” foods, based on the extent and purpose of food processing [6]. Consequently, UPFs refer to those highly profitable formulations made by a series of industrial processes, with a high sensory and commercial appeal [6]. The production of UPFs involves different processing techniques and different ingredients such as sugar, salt, fats, colorings, flavorings, and other additives which are used to imitate the sensory attributes of unprocessed foods [1,11,12], and to make UPFs highly palatable [1]. However, in the NOVA classification, the prefix “ultra-processed” confers food with a negative connotation, referring to what is extremely or excessively processed [10]. Conversely, the NOVA classification has been also criticized based on the great heterogeneity of UPFs in terms of their composition [13].

Consumers generally describe UPFs as highly processed products that often contain additives and artificial and non-natural ingredients, being perceived as industrial foods that undergo chemical and physical processes [2]. Similarly, consumers perceive that these food products have a low nutritional quality and are unhealthful [2]. An increasing body of evidence shows that consumption of UPFs is strongly associated with negative health outcomes, including obesity, cardiovascular diseases and all-cause mortality [14,15] despite consumers still demanding and consuming these food products in their daily routines.

To establish the dimensionality of UPFs’ acceptance construct, we started with the consumers’ conceptualization of UPFs from the critical study of [2]. This was followed by an intent to understand the meaning of UPFs among young consumers [12,16,17] that refers to the multidisciplinary perspective about naturalness and healthiness in UPFs and the factors influencing their purchase and consumption in households with children. Furthermore, other dimensions were added to arrive at a list of nine potential UPF dimensions: (1) product perceived quality; (2) time-saving; (3) affordability; (4) effortlessness; (5) convenience; (6) hedonism; (7) marketing strategies; (8) purchase intention; and (9) satisfaction. We discuss each dimension below, linking it to the UPF acceptance dimensions.

#### 2.1.1. Product Perceived Quality

The concept of product quality can be analyzed from two different perspectives, namely objective quality and perceived quality. On the one hand, objective quality refers to the measurable and verifiable nature of the products, while perceived quality refers to the consumers’ global value or subjective perceptions of quality [18]. Accordingly, food quality is a subjective concept related to the perceived quality of food, which depends on the consumer’s ability to evaluate it. To evaluate the quality of food products, consumers need information on the quality characteristics and the intrinsic attributes of the given product, such as appearance, color, flavor or product presentation [18].

Regarding UPFs, consumers understand that these products are unhealthful [12] because these food products have poor nutritional value and contain excessive fat, sugar, sodium, artificial ingredients and additives [2]. However, the quality characteristics of UPFs may not be familiar to consumers, especially young consumers who may have been less exposed to food information [12], thus distorting their perception of the UPFs’ quality.

#### 2.1.2. Time-Saving

According to [19], this dimension refers to saving time at different stages of the food preparation and consumption process and could be defined as the degree to which a consumer is inclined to save time in regard to meal shopping and meal preparation. This variable is also associated with time pressure in terms of buying and preparing food, and with consumers who have relatively less time available for buying and preparing food.

Therefore, consumers who strongly value saving time when cooking or preparing meals will have a greater propensity towards UPFs [20] since consumers acknowledge that food processing significantly reduces preparation and cooking time [16]. As a result, some consumers feel that consuming UPFs is more adequate when pressed for time for meal planning or meal preparation [17].

#### 2.1.3. Low Price/Affordability

Price is a primary determinant of food demand [21], and a crucial determinant of the UPFs’ consumption [22], since they are relatively low-cost, thus providing a remarkable ability to fit into the family budget compared to meals prepared from scratch [17]. So, a key factor leading consumers to replace traditional food with UPFs is affordability and cheap price [23,24]. At this point, some authors highlight that the low price and affordability of UPFs are due to the use of low-cost ingredients and food additives that reduce calorie prices [24]. Similarly, recent advances in food processing and technology have resulted in greater affordability and ample availability of UPFs [2,25].

#### 2.1.4. Effortless

Previous studies have reported that consumers demand minimizing the physical and mental effort associated with meal planning and preparing and cooking food products [26]. So, the effortless dimension is closely related to food products that help consumers minimize the physical and mental effort required for food preparation, consumption, and cleanup [19,27]. In other words, UPFs save effort in preparation, consumption, and cleanup. Accordingly, avoiding spending effort on meal preparation and clearing up after meals is a strong motive for the consumption and demand of UPFs [17,28].

#### 2.1.5. Convenience

Convenience is a multidimensional concept that could be defined as the time and effort associated with buying or using a product, or that reduces the non-monetary price of a product [29,30]. Accordingly, convenience food products are bought and consumed to save time and effort [27,31]. Further, convenient food products are related to aspects of food preparation such as ease of acquisition, serving, eating and storage [32], and with food whose preparation is fast [27]. Consequently, consumers’ lack of time, knowledge, skills and abilities to prepare home meals influences consumers’ food choices in the direction of convenience food [19,30].

Convenience explains to a greater extent the purchase and consumption of UPFs [33]. When purchasing UPFs, some consumers value that these foods offer great convenience at the time of consumption, since UPFs can be prepared anytime, as well as convenient packaging and long shelf-life, allowing these foods to be stored and consumed anytime [17].

#### 2.1.6. Hedonism

Consumers are often influenced in their food purchase decisions by hedonic values or by the “sensory appeal” of food, which is related to food products’ appealing smell, taste or appearance [34]. Accordingly, prior studies indicate that consuming UPFs is often associated with pleasure, and consumers link their consumption to the pleasant sensory characteristics and hedonic responses and outcomes [35]. So, UPFs are closely associated with the pleasure derived from food consumption, being perceived as “a pleasure to eat” [36].

Likewise, consumers’ increasing demand for UPFs is associated with their high palatability [6] and increased hedonic potential [37]. Prior research suggests that these products are highly palatable due to industrial aromas, free sugars, taste exhausters, sodium, coloring, texturizing agents and unhealthy fats [12,36]. The high palatability and hedonic nature of ultra-processed food products increases the motivation to consume these products despite negative long-term health consequences [38]. However, the hedonistic nature of food products is also related to products that look good, have a good/attractive texture and have a nice appearance, since the sensory attributes of food products influence the consumption of hedonic food products [10].

#### 2.1.7. Marketing Strategies

Marketing strategies and actions describe various product characteristics to make products more salient in consumers’ minds and influence product recognition, food preferences, and eating behavior [35]. Prior research highlights that the food industry aggressively markets UPF products to encourage consumers to consume more frequently and purchase large volumes of these products [2,33,36].

Food companies use many marketing strategies to induce the consumption and sales of UPF products. In the first place, food companies often develop product placement in store-salient locations and other marketing strategies carried out at the point of sale. These marketing actions include ultra-processed food product price discounts on large packages, prominent large displays at the end of the supermarket aisles and placing food products at eye level in highly visible locations or close to cash registers [17,39,40]. In the second place, food companies frequently adopt price strategies, like sales promotions, price rewards and/or periodic price reductions, which could lead to a rise in the choice of UPF products [41,42]. Finally, marketing strategies could be applied to food labels and food labeling design to convey the idea that UPF products are healthful [43], including nutrition marketing claims related to product formulation or production, the presence of specific ingredients or their nutrient content [44,45].

#### 2.1.8. Purchase Intention

The purchase intention could be defined as the tendency to purchase specific brands or products routinely [46] or as reflecting the predicted behavior of the consumer in the more immediate purchasing decision, i.e., what product or brand they will buy on a following occasion [47]. Regarding the purchase decision process for convenience and UPF products, this decision does not only depend on rational processes but also on other factors such as the repetitive purchase behavior of food [42]. More precisely, the purchase intention of ultra-processed food products was found to be negatively related to cooking enjoyment, food involvement and food variety seeking, while it was positively associated with work overload [19], time pressure or value for money [48]. Finally, other factors such as consumer age, education level, working status, nutrition knowledge and cooking skills are significant predictors of the purchase intention of UPF products [27].

#### 2.1.9. Satisfaction

Consumer satisfaction could be defined as a fulfillment response, understood as “the consumer judgment of the product or service feature, or as judgment on the product or service itself” [49]. Further, the generation process of customer satisfaction can be explained by the Expectancy–Disconfirmation Theory [49] which proposes that customers develop expectations about a product before purchasing and subsequently compare the previous product expectations with the actual product performance. Hence, customer satisfaction could be considered a cognitive evaluation of the product’s real performance, compared with the initial consumer expectations. So, satisfaction arises when the expectative is confirmed, while dissatisfaction originates from the expectative disconfirmation. In addition, previous studies have conceptualized consumer satisfaction as either a cognitive response [50], an affective response [51], or an overall evaluative judgment about a product or service [52].

## 3. Empirical Research Design

**Figure 1:**
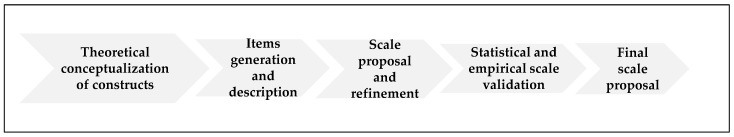
Scale development and validation procedure.

The present study followed the guidance of scale development proposed by [53], focused on the understanding of the latent variables or constructs and the process of construct reliability and validity, and the proposal of [54] focused on the assessment and understanding of the items of the scale. The development and validation of measurement scales comprise five stages (Figure 1).

The first step in proposing and developing a measurement scale is understanding the key concepts associated with UPF acceptance and consumption. For this purpose, in order to carry out the item development an exploratory survey was conducted to evaluate the research items. The items were included in a survey based on the recommendation of five experts in this field of knowledge. More precisely, five academics from the field of consumer behavior and food marketing participated in the initial item development process, and after the experts’ discussion, the scale was constructed covering the dimensions shown in Table 1.

Subsequently, in order to evaluate the content validity, a second group of five experts was selected to review the items. These experts—who were academics in this field of knowledge—examined whether these initial measures and constructs were well constructed. Then, the experts evaluated the relevance of each dimension and its item scale in a discussion group. Finally, these experts evaluated the dimensions based on their experience in terms of how well the dimensions capture the key concepts of acceptance and consumption of UPFs. As a result, a version of the scale was created where the constructs or variables were grouped into nine dimensions (Table 1). Then, the measurement scale was refined, and its validity and reliability were tested. Accordingly, a final scale was proposed and discussed, considering its aim of measuring the acceptance and consumption of UPF food products.

### 3.1. Sampling and Fieldwork

The fieldwork was conducted in Spain in April 2023. Research data were gathered through an online web-based self-administered structured questionnaire designed through Qualtrics software 3.2.0 (see Supplementary Materials). Then, the research questionnaire was distributed among consumers on a random basis, inviting them to participate in the study. Ethical issues do not arise in the present study, since questionnaires were completely anonymous, and all the participants consented to participate in this study for research purposes. The research questionnaire consisted of three sections. In the first section, an introduction was included to explain the primary purpose of the research. This introduction included a conceptualization of UPF products. Then, the second part of the questionnaire was prepared to gather socio-economic and demographic information about the research participants, and the third part included the questions to measure the participant’s assessment of the variables under research. All responses were collected on a seven-point Likert scale ranging from “strongly disagree” (1) to “strongly agree” (7). Finally, a total amount of valid 478 questionnaires was obtained, yielding a sampling error of 4.62% at a confidence level of 95%.

Regarding the sample profile, 51.4% of the respondents were female, while 48.6% were male. Likewise, most participants were between 31 and 40 years old (32.6%), followed by participants aged between 21 and 40 years old (30.5%). Of the participants’ education level, 36.2% have university studies, and 30.6% have secondary education. Concerning the household average income level, 33.4% of the sample reported a monthly household income level of 2200–2700 EUR/month. It was also recorded that 76.7% of the respondents consume UPF products daily, and most (84.2%) consume them frequently.

### 3.2. Data Analysis

A two-step process was developed to examine the validity of the proposed acceptance scale of UPF products. Considering that the scale is a new creation, the first step of the analysis is an Exploratory Factor Analysis (EFA) to determine the subjacent factorial structure [55]. The Maximum Likelihood extraction method will be used since multivariate normality is assumed [56]. Even though the Exploratory Factor Analysis (EFA) helps to determine the dimensionality of the measurement instrument, it only provides evidence of a theoretical factor structure [55]. So, in the second step, a Confirmatory Factor Analysis (CFA) is conducted to analyze the factor structure, and examine construct validity.

## 4. Results

### 4.1. Scale Proposal and Refinement through EFA

Kaiser–Meyer–Olkin (KMO) and Bartlett’s test of sphericity were used to determine the suitability of Exploratory Factor Analysis [55]. The obtained results for the test of Kaiser–Meyer–Olkin (0.974; χ^2^ = 24,686.51; df = 1378), and Barlett’s test of sphericity (<0.05) showed adequate values. Then, the Maximum Likelihood estimation method with Varimax rotation (eigenvalue > 1) Exploratory Factor Analysis (EFA) was conducted to analyze the shared variance among constructs, using the SPSS 28 software. An eigenvalue greater than one was selected as the criterion for determining the number of factors to be extracted, and in the interpretation of the factors, variables with loadings greater than 0.50 were used. The results obtained for EFA extracted nine factors or dimensions, with a total explained variance of 71.32%.

However, one item (MK1) achieved low communalities (<0.50); in turn, this item cannot contribute to the factor structure of the scale [55], being removed. Finally, a total amount of 9 factors and 42 items were retained on the scale. The first factor relates to consumer purchase intention, and contains statements about the future purchase of UPF products. The second factor corresponds to statements related to consumer satisfaction with UPF products. The third factor corresponds to statements connected with food perceived quality, while factor 4 is related to affordable price or a good “value-for-money” relationship with UPF products. Similarly, factor 5 refers to a product that allows consumers to save shopping and meal preparation time. Likewise, factor 6 is related to a product that is mentally and physically efficient in its consumption and preparation, while factor 7 is related to the food product’s hedonic and pleasant aspects, such as being tasty and appealing to the senses. Then, factor 8 includes statements related to the food’s convenience. Finally, factor 9 contains statements about the marketing strategies and actions to induce consumers to purchase ultra-processed food products (Table 2).

Discriminant validity is supported when the 95% confidence interval for the construct correlations does not include 1.0 [57]. Alternatively, when the average variance extracted (AVE) estimates of a given pair of constructs are greater than the square of the construct correlation, discriminant validity is supported. Research findings indicate that AVE values are greater than the squared correlations, thus supporting discriminant validity (Table 3).

### 4.2. Scale Validation through CFA

The measurement scale was conceived by considering latent variables as reflective; and accordingly, the procedure recommended by [55] was adopted to validate reflective constructs. Convergent validity was examined through the Average Variance Extracted (AVE) for each construct, and results indicate that AVE values were above the commonly accepted threshold of 0.50 [55]. Similarly, the analysis of the standardized factor loadings of each item with respect to the latent constructs and Cronbach’s alpha are used to measure the internal consistency of the factors. Cronbach’s alpha values for each construct were above the commonly accepted cutoff point of 0.70 [58]. Then, to evaluate the internal consistency between the items of each construct, Composite Reliability (CR) was used as an indicator, which achieves values higher than 0.70, thus indicating scale validity and internal reliability (Table 4). Finally, the items to be removed from the scale presented loadings below the cutoff point of 0.50, as suggested by [55]; in turn, items Qual1, Price6, Price7, Price8, and Pint4 were removed.

**Figure 2:**
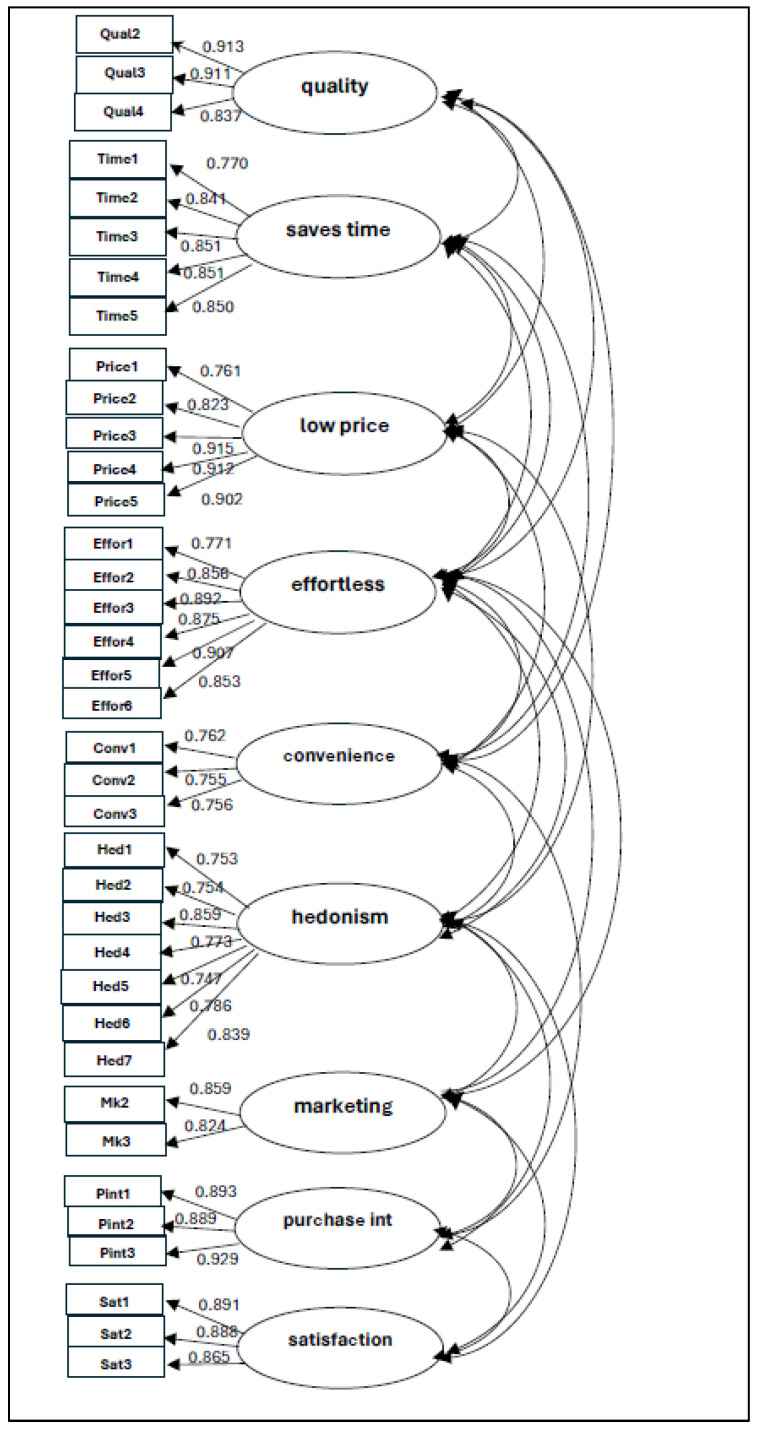
Results of the CFA model.

The construct validity and reliability of the measurement scale were analyzed through Confirmatory Factor Analysis (CFA) using the Amos 28 software. The obtained outcomes showed an adequate model fit for the refined measurement scale: χ^2^/df = 2.208 (*p* < 0.001); CMIN = 1461.493; GFI = 0.861; NFI = 0.922; TLI = 0.950; RMR = 0.054; RMSEA = 0.050; CFI = 0.955, thus achieving adequate values [55]. The obtained results of the CFA analysis show the validity of the proposed measurement scale and report a nine-factor solution including the quality of UPF products, ability to save time, low affordable price and effortless preparation, as well as convenience, hedonic nature, marketing strategies, satisfaction and purchase intention (Table 5). Figure 2 shows the results of the CFA model.

### 4.3. Evaluation of the Nomological Validity

The nomological validity of the measurement scale was examined to evaluate whether the first-order constructs—the quality of UPF products, ability to save time, low affordable price, effortless preparation, convenience, hedonic nature and marketing strategies—could construct the second-order constructs—satisfaction and purchase intention—at a statistical significance level, according to [59]. The results obtained by the Confirmatory Factor Analysis (CFA) confirm that the standardized factor loadings of the first-order constructs achieve values greater than 0.70 at the significance level *p* < 0.05, thus indicating nomological validity [59].

## 5. Discussion

Despite the increasing consumption of UPF products among individuals, no scale was available to measure this phenomenon properly. This is the key motivation of the present research, which develops and validates a scale to measure the consumption and demand of UPF products. Specifically, the present research has developed a scale that quantitatively assesses UPF product acceptance and consumption constructs. The dimensions and scale items were conceptually based on the previous relevant literature. The item development and content validity were examined through an experts’ discussion and then statistically and empirically analyzed through Exploratory Factor Analysis (EFA) and Confirmatory Factor Analysis (CFA). Then, the obtained scale was validated in terms of construct reliability, convergent validity and discriminant validity.

Likewise, regarding the research question of whether it is possible to develop a measurement scale on the acceptance and consumption of UPFs, the answer would be “yes”; further, regarding the research question of which dimensions could be included in this measurement scale, the answer would be “the quality of ultra-processed food products, ability to save time, low affordable price, effortless preparation, convenience, hedonic nature, marketing strategies, satisfaction and purchase intention”. Therefore, the obtained results indicate that the proposed constructs could be considered relevant factors in the acceptance and consumption of UPF products.

### 5.1. Theoretical and Managerial Contributions

The present study yields three theoretical contributions. First, this research has a novelty in developing and validating a scale for measuring the consumption and acceptance of UPF products, being its major theoretical contribution. More precisely, this study is a first attempt to develop an integrative multidimensional scale for the acceptance and consumption of UPF products, since a systematic or valid scale was unavailable before. The second theoretical contribution of the present study is that validating a measurement scale on the acceptance and consumption of UPF products raises relevant questions like what the most relevant items in UPF consumption are or what the main motivations that drive the consumption of UPFs. Finally, the factors or dimensions reported here can be used to assess why individuals demand, consume and accept UPF products, despite other healthier food alternatives being available in the marketplace. In general terms, the 37-question scale allows the researcher to assess consumers’ UPF product’s tendency in applied research in the consumer science area, digging deeper into the food consumers’ attitudes.

Some managerial implications could be derived from the present research. Research findings highlight that food marketers should better understand consumer motivations when demanding and consuming these food products, and this study provides marketers and food companies with a validated instrument to assess consumers’ acceptance and consumption of UPF products. Researchers can also use this scale as a marketing tool to analyze consumer groups and segment the market to develop different marketing strategies targeting different consumer groups. Furthermore, the findings highlight that UPFs can be applied before or after experiencing specific UPF products, thus offering greater flexibility to the researcher.

### 5.2. Research Limitations and Further Research

The present research is not free from limitations. First, the present study proposes and develops a scale for measuring the acceptance and consumption of UPFs as a whole; however, several product categories can be considered UPFs, such as cookies, snacks, instant soup, processed meat, and burgers. Therefore, further studies could consider different product categories, which may influence consumer acceptance and behavior. In the second place, the data utilized in the present study for validation were collected from one country, meaning that the results’ generalizability can be limited. Secondly, considering the increasing growth in UPF consumption, further research is recommended to segment consumers in terms of the benefits sought in ultra-processed food consumption. Furthermore, considering that for scale development research it is recommended to shorten the scale length as much as possible to ensure that interviewees’ engagement is maintained, reducing the length of the UPF scale to less than 37 questions is a challenge that has to be taken into account in future research.

## Figures and Tables

**Table 1:** Dimensions of ultra-processed food acceptance and consumption.

Source	Dimensions	Description of Indicators
[12,18]	Perceived product quality	High or good quality Safety of food products
[19,28]	Saves time	Time-saving in food preparation and cooking Reduces cooking responsibility Reduces time pressure in buying food, preparing meals, food preparation and cooking Ready-to-eat foods
[17,18,24]	Low price/ affordability	Availability and affordability of food products Cheap and low-priced food products
[19,26,28]	Effortless/ Physical and mental effortless	Minimizing physical and mental effort in meal planning and food preparation Requirement of little cooking skills Avoidance of clearing up after meals
[17,19,27,33]	Convenience	Ready-to-eat Easy to prepare, consume, store and preserve Long shelf-life Well preserved Reduction in non-monetary prices of food products
[34,36,37]	Hedonism/pleasure	Tasty, yummy food products Highly palatable food products
[2,33,42,43]	Marketing strategies	Marketing actions on labels (to convey the idea that ultra-processed food products are healthful) Aggressive advertising on social media Sales promotions at the point of sale
[27,42,46]	Purchase intention	Tendency to purchase specific brands/products Repetitive purchase behavior
[49,52]	Satisfaction	Fulfillment response Consumer initial expectations compared to the result obtained Overall evaluative judgment

**Table 2:** EFA for initial measurement items.

Items	Factor 1	Factor 2	Factor 3	Factor 4	Factor 5	Factor 6	Factor 7	Factor 8	Factor 9
Pint3	0.871								
Pint2	0.864								
Pint1	0.810								
Pint4	0.709								
Sat1		0.682							
Sat3		0.670							
Sat2		0.661							
Qual1			0.678						
Qual2			0.675						
Qual3			0.619						
Qual4			0.599						
Price8				0.712					
Price5				0.702					
Price4				0.692					
Price6				0.671					
Price7				0.652					
Price3				0.644					
Price2				0.641					
Price1				0.637					
Time5					0.761				
Time4					0.742				
Time2					0.688				
Time3					0.679				
Time1					0.668				
Effor2						0.719			
Effor3						0.693			
Effor5						0.690			
Effor1						0.682			
Effor6						0.671			
Effor4						0.669			
Ment4						0.662			
Hed2							0.713		
Hed1							0.689		
Hed4							0.675		
Hed7							0.672		
Hed6							0.656		
Hed3							0.656		
Hed5							0.632		
Conv3								0.763	
Conv1								0.761	
Conv2								0.678	
Mk2									0.627
Mk3									0.625

**Table 3:** Correlation coefficients and discriminant validity.

Constructs	Qual	Time	Price	Effort	Conv	Hedon	Mk	Pint	Sat
Qual	**0.899**								
Time	0.540	**0.900**							
Price	0.632	0.656	**0.900**						
Effort	0.640	0.641	0.596	**0.900**					
Conv	0.216	0.350	0.277	0.281	**0.900**				
Hedon	0.457	0.547	0.431	0.438	0.598	**0.900**			
Mk	0.571	0.668	0.682	0.640	0.403	0.580	**0.900**		
Pint	0.494	0.546	0.305	0.506	0.507	0.446	0.433	**0.900**	
Sat	0.677	0.318	0.541	0.624	0.424	0.591	0.603	0.677	**0.900**

Note: the diagonal values in bold represent the square root of the average variance extracted from each construct.

**Table 4:** Validity and reliability of the measurement scale.

Constructs	Items	Standardized Factor Loadings	Cronbach Alpha	CR	AVE
Quality	Qual2	0.913	0.916	0.917	0.788
	Qual3	0.911			
	Qual4	0.837			
Saves time	Time1	0.770	0.921	0.918	0.694
	Time2	0.841			
	Time3	0.851			
	Time4	0.851			
	Time5	0.850			
Low price	Price1	0.761	0.925	0.935	0.742
	Price2	0.823			
	Price3	0.915			
	Price4	0.912			
	Price5	0.902			
Effortless preparation	Effor1	0.773	0.927	0.945	0.740
	Effor2	0.856			
	Effor3	0.892			
	Effor4	0.875			
	Effor5	0.907			
	Effor6	0.853			
Convenience	Conv1	0.762	0.763	0.797	0.566
	Conv2	0.755			
	Conv3	0.756			
Hedonism/ pleasure	Hed1	0.753	0.921	0.920	0.621
	Hed2	0.754			
	Hed3	0.859			
	Hed4	0.773			
	Hed5	0.747			
	Hed6	0.786			
	Hed7	0.839			
Mk strategies	Mk2	0.859	0.828	0.758	0.610
	Mk3	0.824			
Purchase intention	Pint1	0.893	0.930	0.931	0.817
	Pint2	0.889			
	Pint3	0.929			
Satisfaction	Sat1	0.891	0.913	0.913	0.777
	Sat2	0.888			
	Sat3	0.865			

**Table 5:** Proposal of the final scale.

Construct	Code	Items
Quality	Qual2	Ultra-processed food products have a high quality
	Qual3	Ultra-processed food products offer a reliable and trustworthy quality
	Qual4	Ultra-processed food products are safe
Saves time	Time1	Since I am always under time pressure, I try to save time while cooking
	Time2	When eating at home, I prefer to cook and eat meals that can be prepared quickly
	Time3	Preferably, I spend as little time as possible cooking and on meal preparation
	Time4	I try to do my food shopping as quickly as possible
	Time5	I do not like to spend too much time shopping for food
Low price	Price1	Ultra-processed food products are not expensive
	Price2	Ultra-processed food products offer a good price-quality relationship
	Price3	In the food sector, I consider ultra-processed foods a good purchase
	Price4	Ultra-processed food products offer high value compared to their price
	Price5	Ultra-processed food products offer the best quality for the best price
Effortless preparation	Effor1	I do not want to think about what to cook for a long time
	Effor2	I try to minimize the mental effort when cooking and preparing meals
	Effor3	Cooking means mental effort, which I try to avoid if possible
	Effor4	The less energy I need to cook and to prepare a meal, the better
	Effor5	Cooking means physical effort, which I try to avoid if possible
	Effor6	At home, I preferably eat meals that can be prepared quickly
Convenience	Conv1	Ultra-processed food products are convenient and save time
	Conv2	Ultra-processed food products are a good last-minute meal solution
	Conv3	Ultra-processed food products are easy to prepare
Hedonism	Hed1	Ultra-processed food products look nice
	Hed2	Ultra-processed food products are attractive and appealing
	Hed3	Eating ultra-processed food products is a very pleasant experience
	Hed4	Ultra-processed food products taste good
	Hed5	All the senses are involved when eating ultra-processed food products
	Hed6	In general terms, I believe that ultra-processed food products have a pleasant texture
	Hed7	Eating ultra-processed food products is related with pleasant tastes, smells and seeing
Marketing strategies	Mk2	I often purchase ultra-processed food products because they have frequent sales promotions and price reductions
	Mk3	When purchasing ultra-processed food products, information from advertising helps me to make better buying decisions
Purchase intention	Pint1	I would purchase ultra-processed food products in the future
	Pint2	I am likely to purchase ultra-processed food products in the next months
	Pint3	The likelihood that I would purchase ultra-processed food products is high
Satisfaction	Sat1	Ultra-processed food products meet my expectations
	Sat2	When I eat ultra-processed food products, I’m satisfied with the experience
	Sat3	Ultra-processed food products satisfy my needs and desires

## Data Availability

The original contributions presented in the study are included in the article/Supplementary Material, further inquiries can be directed to the corresponding author.

## Supplementary Materials

The following supporting information can be downloaded at: https://www.mdpi.com/article/10.3390/foods13101481/s1, Questionnaire.
