# Supplementary material for: Proposal and Validation of a Measurement Scale of the Acceptance of Ultra-Processed Food Products
Source: Foods. 2024 May 10;13(10):1481. doi: 10.3390/foods13101481 (PMC11120215; doi:10.3390/foods13101481)
Supplement: Supplementary file 1 [file foods-13-01481-s001.zip › foods-2986547-supplementary.pdf]

foods

Supplementary materials

Questionnaire

Construct	Code	Items
**Quality**	Qual1	Ultra-processed food products offer an acceptable quality
	Qual2	Ultra-processed food products have a high quality
	Qual3	Ultra-processed food products offer a reliable and trustworthy quality
	Qual4	Ultra-processed food products are safe
**Saves time**	Time1	Since I am always under time pressure, I try to save time while cooking
	Time2	When eating at home, I prefer to cook and eat meals that can be prepared quickly
	Time3	Preferably, I spend as little time as possible cooking and on meal preparation
	Time4	I try to do my food shopping as quickly as possible
	Time5	I do not like to spend too much time shopping for food
**Low price**	Price1	Ultra-processed food products are not expensive
	Price2	Ultra-processed food products offer a good price-quality relationship
	Price3	In the food sector, I consider ultra-processed foods a good purchase
	Price4	Ultra-processed food products offer high value compared to their price
	Price5	Ultra-processed food products offer the best quality for the best price
	Price6	Ultra-processed food products are affordable for most consumers
	Price7	Ultra processed food products are a good option when you want to get a good value for money
	Price8	I consider ultra-processed food products to be low priced
**Effortless preparation**	Effor1	I do not want to think about what to cook for a long time
	Effor2	I try to minimize the mental effort when cooking and preparing meals
	Effor3	Cooking means mental effort, which I try to avoid if possible
	Effor4	The less energy I need to cook and to prepare a meal, the better
	Effor5	Cooking means physical effort, which I try to avoid if possible
	Effor6	At home I preferably eat meals that can be prepared quickly
**Conven-ience**	Conv1	Ultra-processed food products are convenient and save time
	Conv2	Ultra-processed food products are a good last-minute meal solution
	Conv3	Ultra-processed food products are easy to prepare
**Hedonism**	Hed1	Ultra-processed food products look nice
	Hed2	Ultra-processed food products are attractive and appealing
	Hed3	Eating ultra-processed food products is a very pleasant experience
	Hed4	Ultra-processed food products taste good
	Hed5	All the senses are involved when eating ultra-processed food products
	Hed6	In general terms, I believe that ultra-processed food products have a pleasant texture
	Hed7	Eating ultra-processed food products is related with pleasant tastes, smells and seeing
**Marketing strategies**	Mk1	There is a great assortment and availability of ultra-processed food products in grocery shops and supermarkets
	Mk2	I often purchase ultra-processed food products because they have frequent sales promo-tions and price reductions
	Mk3	When purchasing ultra-processed food products, information from advertising helps me to make better buying decisions
Purchase intention	Pint1	
	Pint2	I would purchase ultra-processed food products in the future
	Pint3	I am likely to purchase ultra-processed food products in the next months
	Pint4	The likelihood that I would purchase ultra-processed food products is high

		I would rather buy ultra-processed food products, even if more healthy options are available
Satisfaction	Sat1	Ultra-processed food products meet my expectations
	Sat2	When I eat ultra-processed food products, I'm satisfied with the experience
	Sat3	Ultra-processed food products satisfy my needs and desires
